# The role of solar radiation and tidal emersion on oxidative stress and glutathione synthesis in mussels exposed to air

**DOI:** 10.7717/peerj.15345

**Published:** 2023-05-11

**Authors:** Daniel C. Moreira, Marcus Aurélio da Costa Tavares Sabino, Marina Minari, Felipe Torres Brasil Kuzniewski, Ronaldo Angelini, Marcelo Hermes-Lima

**Affiliations:** 1 Department of Cell Biology, University of Brasilia, Brasilia, Brazil; 2 Faculty of Medicine, University of Brasilia, Brasilia, Brazil; 3 Department of Civil and Environmental Engineering, Federal University of Rio Grande do Norte, Natal, Brazil

**Keywords:** Aerial exposure, Antioxidant, Bivalve, Hypoxia, Reactive oxygen species, Ultraviolet radiation, *Brachidontes solisianus*, Preparation for oxidative stress, Mytilidae

## Abstract

Preparation for oxidative stress (POS) is a widespread adaptive response to harsh environmental conditions, whose hallmark is the upregulation of antioxidants. In contrast to controlled laboratory settings, animals are exposed to multiple abiotic stressors under natural field conditions. Still, the interplay between different environmental factors in modulating redox metabolism in natural settings remains largely unexplored. Here, we aim to shed light on this topic by assessing changes in redox metabolism in the mussel *Brachidontes solisianus* naturally exposed to a tidal cycle. We compared the redox biochemical response of mussels under six different natural conditions in the field along two consecutive days. These conditions differ in terms of chronology, immersion/emersion, and solar radiation, but not in terms of temperature. Animals were collected after being exposed to air early morning (7:30), immersed during late morning and afternoon (8:45–15:30), and then exposed to air again late afternoon towards evening (17:45–21:25), in two days. Whole body homogenates were used to measure the activity of antioxidant (catalase, glutathione transferase and glutathione reductase) and metabolic (glucose 6-phosphate dehydrogenase, malate dehydrogenase, isocitrate dehydrogenase and pyruvate kinase) enzymes, reduced (GSH) and disulfide (GSSG) glutathione levels, and oxidative stress markers (protein carbonyl and thiobarbituric acid reactive substances). Air and water temperature remained stable between 22.5 °C and 26 °C during both days. Global solar radiation (GSR) greatly differed between days, with a cumulative GSR of 15,381 kJ/m^2^ for day 1 and 5,489 kJ/m^2^ for day 2, whose peaks were 2,240 kJ/m^2^/h at 14:00 on day 1 and 952 kJ/m^2^/h at 12:00 on day 2. Compared with animals underwater, emersion during early morning did not elicit any alteration in redox biomarkers in both days. Air exposure for 4 h in the late afternoon towards evening caused oxidative damage to proteins and lipids and elicited GSH synthesis in animals that had been previously exposed to high GSR during the day. In the following day, when GSR was much lower, exposure to air under the same conditions (duration, time, and temperature) had no effect on any redox biomarker. These findings suggest that air exposure under low-intensity solar radiation is not sufficient to trigger POS in *B. solisianus* in its natural habitat. Thus, natural UV radiation is possibly a key environmental factor that combined to air exposure induces the POS-response to the stressful event of tidal variation in this coastal species.

## Introduction

Many animals upregulate their endogenous antioxidant systems when exposed to adverse environmental conditions. This kind of biochemical adaptation was originally called ‘preparation for oxidative stress’ (POS) and proposed to be a mechanism that allows animals to deal with the extremes of the environment, especially conditions leading to metabolic depression and/or low oxygen availability (Giraud-Billoud et al., 2019; Moreira et al., 2023; Rodriguez, Campoy-Diaz & Giraud-Billoud, 2023). The POS mechanism was reported to work in more than 80 animal species when exposed to freezing, hypoxia or anoxia, severe dehydration, and air (water breathing animals), as well as during estivation (Moreira et al., 2021a). In addition, the POS phenotype was also documented for eight animal phyla, indicating its broad relevance in the animal kingdom (Moreira et al., 2017b). The molecular mechanism underlying POS involves activating redox-sensitive transcriptional factors by reactive oxygen/nitrogen species (RONS), which increase the expression of endogenous antioxidants (Hermes-Lima et al., 2015).

Since almost all POS-related studies, until 2016, were performed in the lab, we proposed a challenge to show more evidence of POS phenotype in animals under natural conditions, *i.e.,* in the wilderness (Moreira et al., 2017b). Several studies met this challenge, including the investigation of fish endurance in a seasonal hypoxic stream (Ondei et al., 2020), frog estivation in a semi-desertic region (two species; Moreira et al., 2020; Moreira et al., 2021a, toad hibernation in cold mountains in India (Patnaik & Sahoo, 2021), and aerial exposure of mollusks *Brachidontes solisianus* and *Littorina kurila*during the low tide (Istomina, Belcheva & Chelomin, 2013; Moreira et al., 2021b). We also recently highlighted that ultraviolet radiation (UVR) could trigger a POS-response in several invertebrate species with a molecular mechanism similar to hypoxia-induced POS (Geihs et al., 2020). Under certain conditions, UV exposure can trigger RONS formation, which may cause an increase in the activity and/or expression of endogenous antioxidant enzymes –this is the hallmark of the “regular” POS mechanism.

Considering the recent inclusion of UVR as a trigger of the POS phenotype (Geihs et al., 2020), we decided to investigate whether solar radiation could modulate the redox metabolism in mussels *B. solisianus* in the natural environment. We recently demonstrated the activation of the glutathione metabolism in these mussels during aerial exposure cycles in the wild (Moreira et al., 2021b), but without considering any possible influence of solar UVR. Moreover, there are many studies in the literature demonstrating that hypoxia or aerial exposure in bivalves induce the POS phenotype in laboratory-controlled studies (Almeida et al., 2005; David et al., 2005; Chen et al., 2007a; De Zoysa et al., 2009; Hu et al., 2015; Giannetto et al., 2017), but none of these works investigated the possible effects of UVR. Thus, in the present work, we explored possible interactions between solar radiation and air exposure on the POS response of *B. solisianus* under natural conditions –in the same environment as our previous field study with this species (Moreira et al., 2021b). We postulated that animals exposed to different solar radiation levels would respond differently to the stress of being air-exposed during a low tide. To analyze this, we investigated animals exposed to air or underwater in low or high solar radiation incidence conditions. All animals were collected under very similar air and seawater temperatures. Then, we measured the levels of glutathione, antioxidant enzymes, metabolic enzymes, and oxidative stress markers in these bivalves. Previously published data (Moreira et al., 2021b) were compared with the new results on mussel response to aerial exposure and solar radiation.

## Materials & Methods

### Fieldwork

**Figure 1:**
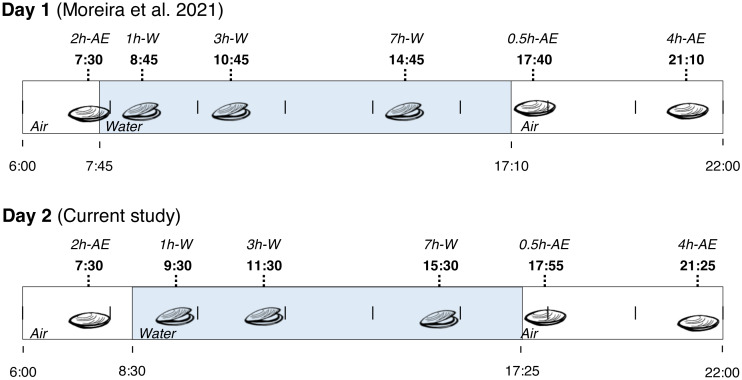
Collection time-periods, time on which animals were exposed to air or underwater, experimental groups and duration of immersion or emersion of *Brachidontes solisianus* mussels during the tidal cycle. Animals were exposed to air twice (early morning and late afternoon) on each day in a total of four air exposure events at a rocky shore in the city of Penha, Santa Catarina State, in southern Brazil (−26.7868S, −48.6051W). Blue shading represents the period that tide submerged mussels at the collection site, when mussels remained with their valves open.

Fieldwork was conducted in a rocky shore in the city of Penha (Santa Catarina State, Brazil) at 26°47′12.2″S, 48°36′18.2″W. On November 2nd (2014), adult mussels (*Brachidontes solisianus*, Bivalvia: Mytilidae) weighting 0.2–0.4 g were collected from a single rock at several time-points from 7:30 a.m. to 9:25 p.m. During this period the animals were exposed to air for at least 2 h (>2 h-AE group), submerged for 7 h (1 h-W, 3 h-W, and 7 h-W groups), and then exposed to air again for 4 h (0.5 h-AE and 4 h-AE groups). In summary, mussels were exposed to air twice: first in the early morning (with very little exposure to sunlight) and then in the evening (after a whole day of sunlight exposure). Animals were taken from the rock by hand, quickly cleaned and flash frozen in liquid N_2_ on site. At the end of the day, the frozen specimens were transported to the laboratory and stored at −80 °C. This study was conducted under the license issued by ICMBio and SISBIO (permit number 20030–9). The experimental groups that were collected on November 2nd, 2014, were exactly the same experimental groups collected on the day before (November 1st, Fig. 1), whose biochemical data have already been published (Moreira et al., 2021b). The site of collection was exactly the same in both days and each individual mussel was an experimental unit, with an N of 3–11 per experimental group/variable (all non-enzymatic redox variables presented N ≥7).

Sampling was done on the same beach-rock with a height of approximately 2 ft. During the emersion periods, mussels were completely exposed to air and presented closed valves. In contrast, their shell valves were clearly open when underwater. Collected bivalves when underwater down to 1 to 2 ft from the surface. During both collection days, seawater was transparent down to the depth and location where mussels were collected. Water transparency in the site, evaluated by the Secci disk, ranges from 5 to 12 ft along all year long (Resgalla et al., 2007). Furthermore, *B. solisianus* are vertically oriented on the substrate, perpendicularly to the surface, which favors solar radiation reaching the shells’ interior despite the narrow aperture. The shells for adults of this species are fully opaque. Thus, solar radiation cannot reach the internal organs when shut (Abbott, 1974; Ruppert, Fox & Barnes, 2004; Monteiro-Ribas et al., 2006).

In addition to the recording of seawater and air temperatures on site using a mercury thermometer, global solar radiation (GSR) was determined with a pyranometer (CM6B, Kipp & Zonen, Delft, Netherlands) located at the Itajaí-A868 weather station from the National Meteorological Institute (INMET), 25 km from the mussel collection site. GSR strongly relates to solar UVR, and cloudy periods present significantly smaller GSR, with proportionally less UVR when compared with sunny periods (Porfirio et al., 2012; Wang et al., 2013). We calculated the cumulative global solar radiation by summing hourly GSR up to the respective hour.

Mussel collection happened on a clear sky day on November 1st, 2014, and a cloudy day on November 2nd. Thus, days 1 and 2 of collection are also called “sunny” and “cloudy”. Thus, the following groups were formed for each day: >2 h-AE group, 1 h-W, 3 h-W, 7 h-W, 0.5 h-AE, and 4 h-AE; a total of 12 experimental groups (six collections points ×2 days). For comparison purposes, the group of mussels underwater for 7 h was appointed as the “control” group.

### Antioxidant and metabolic enzymes

Mussels were individually weighted and then pulverized in liquid nitrogen. The pulverized sample was transferred to a glass tissue homogenizer (Tenbroeck type) containing 0.1 M Tris (pH 7.6), 5 mM EDTA, 1 mM phenylmethylsulfonyl fluoride and 1:1,000 (v/v) protease inhibitor cocktail (P8340; Sigma Aldrich, St. Louis, MO, USA). After homogenization, samples were centrifuged at 10, 000 × g for 15 min at 4 °C and the supernatants were collected for the assessment of enzymatic activities. The activities of catalase, glutathione transferase (GST), glutathione reductase (GR), glutathione peroxidase (GPX), glucose 6-phosphate dehydrogenase (G6PDH), pyruvate kinase (PK), malate dehydrogenase (MDH) and isocitrate dehydrogenase (IDH) were measured using spectrophotometric kinetic assays as described in Moreira et al. (2021b). The activities of opine dehydrogenase (ODH), strombine dehydrogenase (SDH), and alanopine dehydrogenase (ADH) were also measured using spectrophotometric kinetic assays, as described in Schiedek (Schiedek, 1997).

The activity of all enzymes was expressed as U per milligram of protein in the supernatant, which was measured using Coomassie brilliant blue G-250 and a standard curve built with bovine serum albumin as standard (Bradford, 1976).

### Glutathione and oxidative stress markers

For the measurement of reduced (GSH), disulfide (GSSG) and total glutathione (tGSH = GSH + 2 ×GSSG) concentrations, as well as thiobarbituric acid reactive substances (TBARS) and protein carbonyl levels, mussels were individually weighted and then pulverized in liquid nitrogen. The pulverized sample was transferred to a glass tissue homogenizer (Tenbroeck type) containing trichloroacetic acid (TCA) and homogenized. The crude homogenate was used to measure TBARS levels as described by Buege & Aust (1978). Another set of homogenates was centrifuged at 10,000 × g for 6 min at 4 °C; the supernatants were used to measure GSH, GSSG, and tGSH levels in the enzymatic recycling method (Tietze, 1969; Griffith, 1980) with modifications (Welker, Moreira & Hermes-Lima, 2016), and the pellets were used to measure protein carbonyl content as described by Fields & Dixon (1971). Additional details can be found in Moreira et al. (2021b).

### Statistics

Before any statistical analysis, we compiled the original data generated in the present study (day 2) with those already published in a similar study (day 1, Moreira et al., 2021b). The reasons are: the experimental groups (time of air exposure and time underwater) in the two datasets are the same; the animals were collected from the very same rock; small variability in the moment of animal collection (∼10 min; Fig. 1). First, data were tested for normality by group. Secondly, UVR and temperature values on days 1 and 2 were compared using t-tests. In order to compare within animals’ groups, a two-way ANOVA was used for each variable using two factors: day and time under condition (underwater or exposed to air). For glutathione’ parameters, we compared air-exposed groups (“>2 h-AE”, “0.5 h-AE” and “4 h-AE”) and underwater animals (“1 h-W”, “3 h-W” and “7 h-W” groups combined, yielding the large group “1,3,7 h-W”). Considering that glutathione results from the three underwater groups were unchanged, the clustering simplifies comparisons, increases the number of underwater replicates, and is more representative of underwater “control” animals. When necessary, we performed a Tukey’s test. Two-way ANOVAs were also used for data on enzymatic activity (catalase, GST, GR, and G6PDH), comparing air-exposed (“0.5 h-AE” and “4 h-AE”) with underwater mussels (“3 h-W” and “7 h-W” combined as “3,7h-W”) in sunny and cloudy days. Two-way ANOVA tests were also employed for metabolic enzymes, TBARS, and protein carbonyl levels, comparing air-exposed with underwater animals on both days.

In addition to the biochemical data of animals collected on November 2nd (day 2), we are also presenting novel data on global solar radiation on both days and enzymatic activity of enzymes involved in anaerobic metabolism in animals collected on November 1st (day 1) and November 2nd (day 2).

## Results

### Environmental data

**Figure 2:**
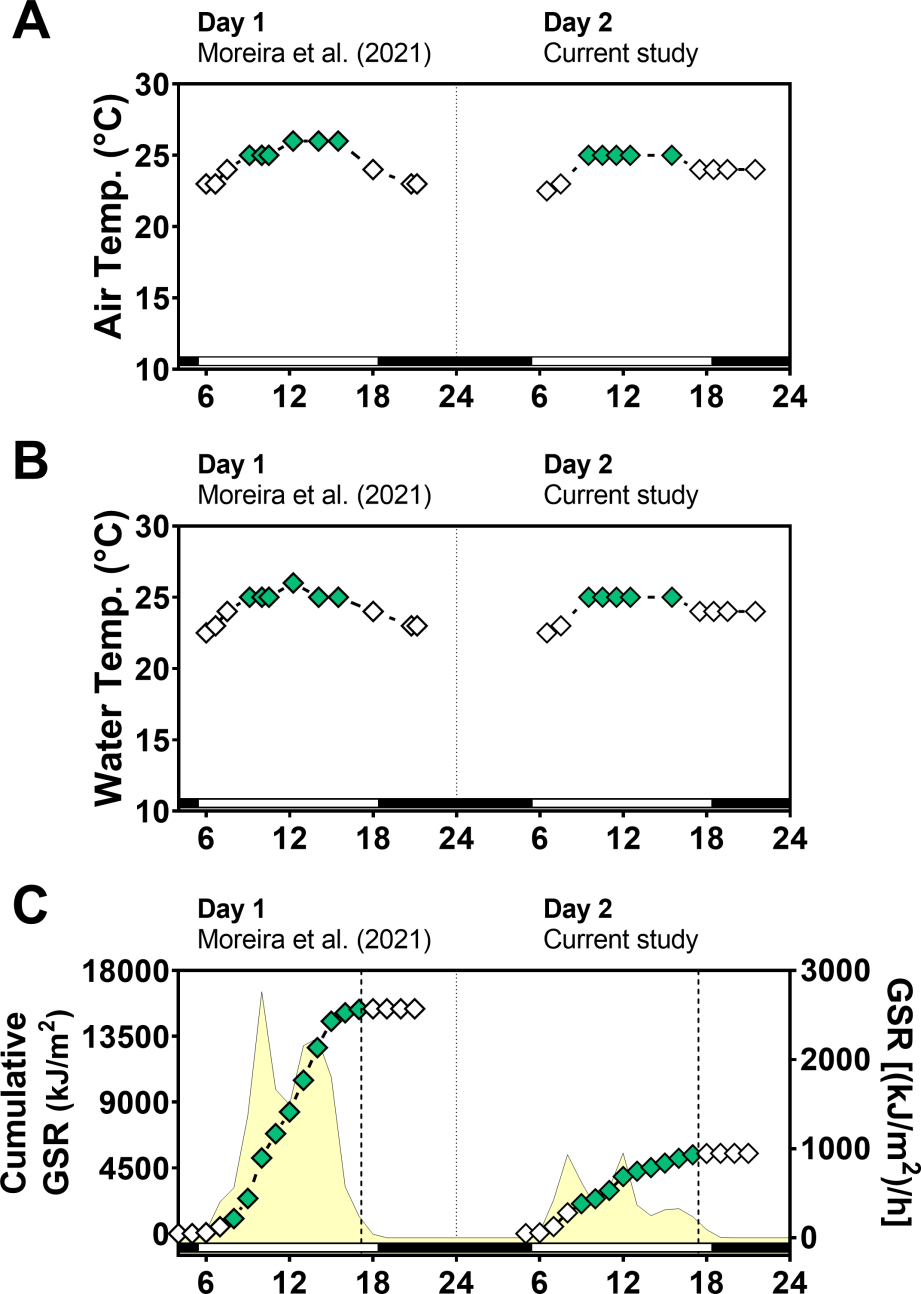
Temperature and global solar radiation on both days of animal collection. (A) Air temperature (° C). (B) Seawater temperature (° C). (C) Cumulative global solar radiation (kJ/m ^2^). Hourly global solar radiation [(kJ/m ^2^)/h] is plotted on the right *y*-axis. Solar radiation was measured with a pyranometer (model CMB4) from the National Meteorological Institute (INMET). Day 1 (Novembe-r 1st, 2014) had high solar radiation intensity and Day 2 (November 2nd, 2014) was cloudy and had low solar radiation intensity. Solar radiation differs significantly between days (paired *t* test = 3.012, *df* = 12, *p* < 0.0102).

Global solar radiation (GSR) was higher in day 1 (sunny) (mean: 1,183 kJ/m^2^/h) than in day 2 (cloudy) (mean: 422 kJ/m^2^/h) (paired *t*-test = 3.0, *p* = 0.0102) with variations along the day (Fig. 2). Total cumulative GSR was 15,381 kJ/m^2^ for day 1 and 5,489 kJ/m^2^ for day 2. Air and water temperature values ranged narrowly between 23 °C and 26 °C and were similar in the two days between 6:00 and 21:30 (air temperature *t*-test = 0.42, *p* = 0.67 and water temperature *t*-test = 0.04, *p* = 0.96).

### Glutathione

**Figure 3:**
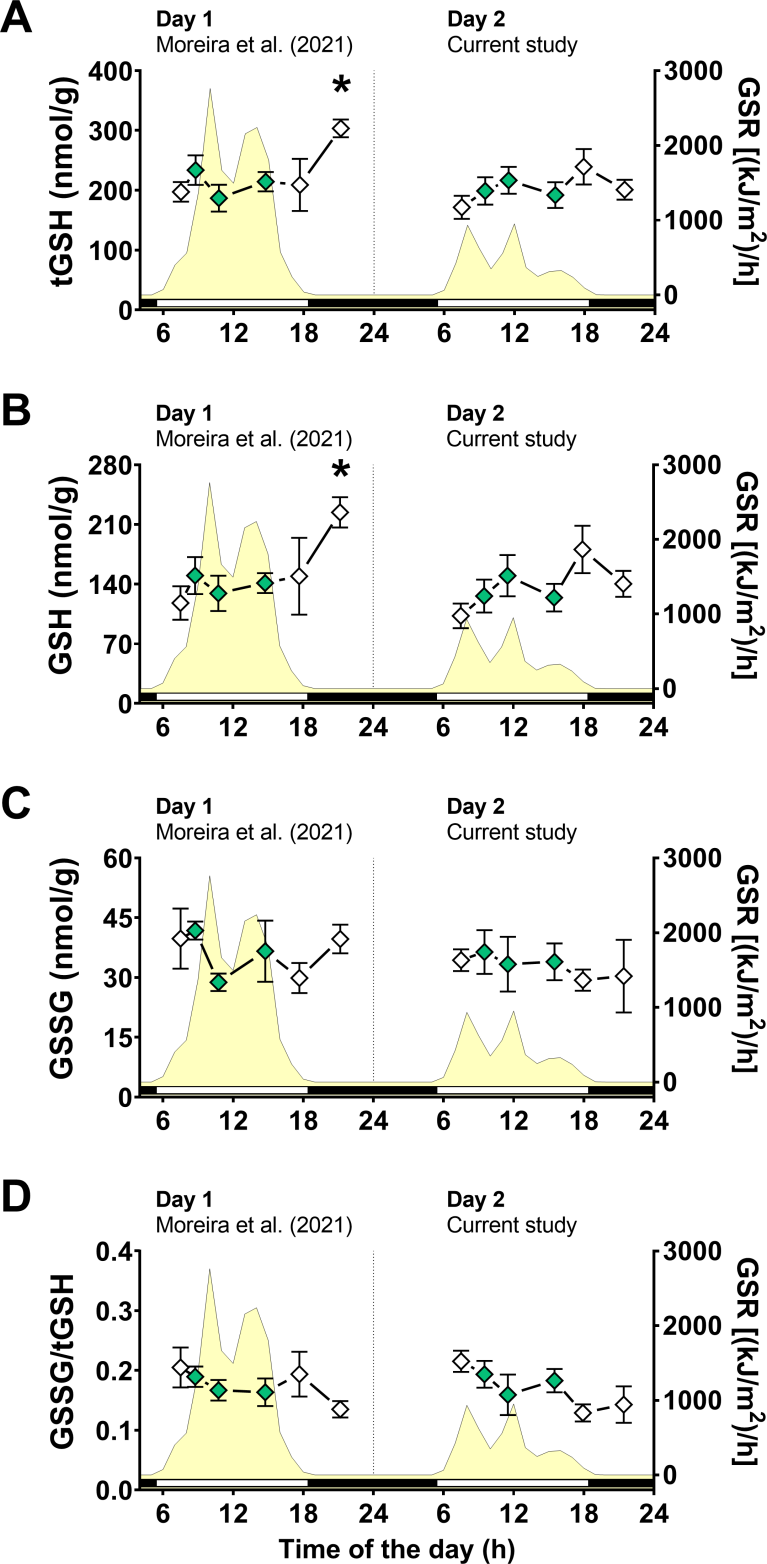
Glutathione levels in *Brachidontes solisianus* mussels during the tidal cycle on two different days. Animals were exposed to air twice (early morning and late afternoon) on each day in a total of four air exposure events. (A) Total glutathione (tGSH) levels, (B) reduced glutathione (GSH) levels, (C) disulfide glutathione (GSSG) levels, and (D) GSSG/tGSH ratio. The yellow shading represents global solar radiation on the right *y* axis. The black and white bars denotes the photoperiod. Blank diamonds represent animals exposed to air and green diamonds represent those under water. The asterisk denotes significant difference between animals exposed to air for 4 h and those underwater for 1, 3, and 7 h combined. N = 7–11. Data for animals collected on day 1 were retrieved from Moreira et al. (2021b) and aggregated with those of the current study before statistical analysis. Data are shown as mean ± standard error.

Following the same approach on day 1 (Moreira et al., 2021b), we first measured glutathione levels, as total glutathione (tGSH), reduced glutathione (GSH), disulfide glutathione (GSSG), and disulfide/total ratio (GSSG/tGSH). Two-way ANOVA tests revealed that air exposure had a significant effect on tGSH and GSH levels, whereas the day of animal collection had no significant effect (File S1). Moreover, a significant interaction between the effects of air exposure and day of collection on both tGSH and GSH was detected (File S1), indicating that the effect of air exposure on these variables depended on the day of collection. In other words, since the two days differed mainly in terms of solar radiation incidence, the effect of air exposure on tGSH and GSH depended on the degree of radiation exposure. Multiple comparisons analyses identified that the concentrations of tGSH (Fig. 3A) and GSH (Fig. 3B) in mussels exposed to air for 4 h (4 h-AE) in day 1 were significantly different from those in mussels underwater (1, 3, 7 h-W) in either day 1 or day 2 (File S1). Levels of tGSH were also higher in animals exposed to air for 4 h in day 1 *versus* those exposed to air for 4 h in day 2 (File S1) The average increase after 4 h of air-exposure for the two glutathione variables (GSH and tGSH) in relation to the underwater group was ∼1.6 fold.

A two-way ANOVA showed no statistically significant interaction between the effects of air exposure and day of collection, as well as no simple main effect of these factors, on GSSG levels (Fig. 3C; File S1). In the case of GSSG/tGSH (Fig. 3D), a significant effect of air exposure was detected (File S1). However, multiple comparisons tests did not detect significant differences between experimental groups. No significant effect of day of collection or the interaction between day and air exposure on GSSG/tGSH was detected (File S1).

### Antioxidant enzymes and oxidative stress markers

**Figure 4:**
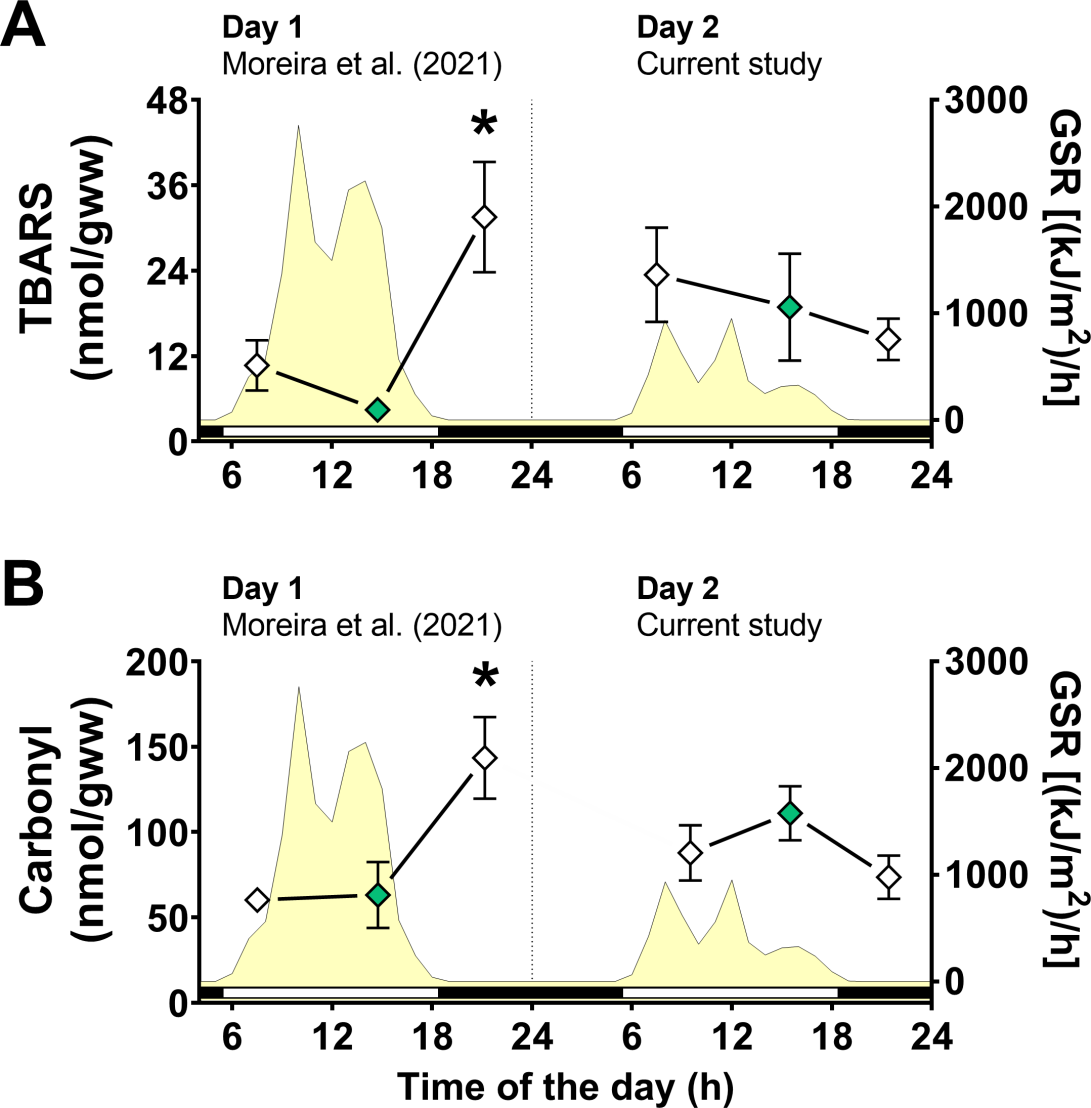
Oxidative stress markers in *Brachidontes solisianus* mussels during the tidal cycle on two different days. Animals were exposed to air twice (early morning and late afternoon) on each day in a total of four air exposure events. (A) Thiobarbituric acid reactive substances (TBARS) content, a marker of lipid peroxidation. (B) Protein carbonyl content, a marker of oxidatively damaged proteins. The yellow shading represents global solar radiation on the right y axis. The black and white bars denotes the photoperiod. Blank diamonds represent animals exposed to air and green diamonds represent those under water. The asterisk denotes significant difference between animals exposed to air for 4 h and those underwater for 1, 3, and 7 h combined. N = 8–9. Data for animals collected on day 1 were retrieved from Moreira et al. (2021b) and aggregated with those of the current study before statistical analysis. Data are shown as mean ±  standard error.

Next, we focused on fewer groups to investigate any changes in the activity of antioxidant enzymes (Table 1) or oxidative stress markers (Fig. 4). Two-way ANOVA tests did not reveal any significant effect of day of collection, air exposure or interaction between them on catalase, GST, and G6PDH activities (File S1). A significant interaction between the effects of day of collection and air exposure on GR activity was detected, but multiple comparison tests did not reveal any differences between groups (File S1).

**Table 1:** Enzymatic activity of antioxidant and related enzymes in *Brachidontes solisianus* mussels underwater for 3 or 7 h and exposed to air for 0.5 or 4 h during the tidal cycle. Values are mean ± standard error, and (*N* = number of organisms).

**Antioxidant** **enzyme**	**Experimental** **group**			
	3h-W	7h-W	0.5h-AE	4h-AE
*Day 1*				
Catalase (U mg^−1^)	229.61 ± 8.08 (6)	239.93 ± 45.33 (6)	257.17 ± 31.89 (6)	223.50 ± 28.49 (9)
GST (mU mg^−1^)	268.28 ± 23.63 (6)	241.21 ± 24.29 (6)	286.31 ± 34.77 (6)	250.01 ± 29.75 (6)
GR (mU mg^−1^)	24.92 ± 2.60 (6)	23.45 ± 2.72 (6)	31.64 ± 3.71 (6)	25.13 ± 3.95 (7)
G6PDH (mU mg^−1^)	14.78 ± 2.88 (5)	18.15 ± 3.06 (6)	21.39 ± 1.77 (6)	13.56 ± 2.80 (7)
*Day 2*				
Catalase (U mg^−1^)	273.11 ± 27.06 (7)	214.66 ± 29.35 (5)	247.89 ± 27.22 (6)	193.29 ± 26.89 (6)
GST (mU mg^−1^)	239.77 ± 23.90 (5)	324.61 ± 30.06 (5)	241.72 ± 8.52 (5)	170.23 ± 29.98 (5)
GR (mU mg^−1^)	29.23 ± 2.45 (5)	25.13 ± 2.38 (5)	19.37 ± 0.51 (5)	25.30 ± 4.56 (5)
G6PDH (mU mg^−1^)	19.33 ± 3.29 (6)	10.62 ± 1.25 (5)	13.00 ± 1.59 (5)	18.61 ± 4.74 (5)

**Notes.**

GST glutathione transferase GR glutathione reductase G6PDH glucose 6-phosphate dehydrogenase

Data for Day 1 are from Moreira et al. (2021b).

A significant interaction between the effects of day of collection and air exposure on TBARS content was detected (File S1) and multiple comparison tests indicated that animals exposed to air for 4 h on day 1 had higher TBARS levels than those in mussels underwater for 7 h on day 1 (Fig. 4A). Similarly, the effects of day of collection and air exposure on carbonyl protein levels had a significant interaction (File S1). Multiple comparisons tests revealed that carbonyl protein levels in mussels exposed to air for 4 h were higher than those in mussels underwater for 7 h on day 1 and mussels exposed to air for 4 h on day 2 (Fig. 4B).

### Metabolic enzymes

There was no significant effect of day of collection, air exposure or interaction between them on the activities of intermediary metabolism enzymes (Table 2) and anaerobic metabolism enzymes (Table 3; File S1).

**Table 2:** Activities of metabolic enzymes in *Brachidontes solisianus* mussels underwater for 7 h and exposed to air for 4 h during the tidal cycle. Values are mean ± standard error, and (*N* = number of organisms).

**Metabolic enzyme**	**Experimental** **group**	
	7h-W	4h-AE
*Day 1*		
Malate dehydrogenase (mU mg^−1^)	1,393.32 ± 210.48 (6)	1,273.67 ± 210.98 (7)
Isocitrate dehydrogenase (mU mg^−1^)	45.29 ± 5.88 (6)	51.02 ± 5.67 (6)
Pyruvate kinase (mU mg^−1^)	124.12 ± 17.31 (4)	95.96 ± 17.95 (6)
*Day 2*		
Malate dehydrogenase (mU mg^−1^)	1,281.11 ± 203.25 (5)	1,255.29 ± 245.89 (6)
Isocitrate dehydrogenase (mU mg^−1^)	47.71 ± 4.22 (5)	48.09 ± 10.56 (5)
Pyruvate kinase (mU mg^−1^)	173.33 ± 87.86 (4)	129.61 ± 26.95 (6)

**Notes.**

Data for Day 1 are from Moreira et al. (2021b).

**Table 3:** Activities of anaerobic metabolism enzymes in *Brachidontes solisianus* mussels underwater for 3 or 7 h and exposed to air for 0.5 or 4 h during the tidal cycle.

**Enzyme**	**Experimental** **group**			
	3h-W	7h-W	0.5h-AE	4h-AE
*Day 1*				
Opine dehydrogenase (mU mg^−1^)	24.93 ± 3.50 (3)	21.14 ± 5.90 (3)	39.50 ± 3.26 (3)	27.40 ± 6.67 (5)
Strombine dehydrogenase (mU mg^−1^)	96.96 ± 9.75 (3)	98.99 ± 3.38 (3)	106.72 ± 12.91 (3)	89.02 ± 10.27 (4)
Alanopine dehydrogenase (mU mg^−1^)	285.08 ± 81.47 (4)	234.60 ± 45.62 (6)	301.03 ± 66.48 (4)	269.45 ± 24.37 (5)
*Day 2*				
Opine dehydrogenase (mU mg^−1^)	39.51 ± 11.25 (4)	17.31 ± 2.92 (3)	36.40 ± 7.47 (4)	20.46 ± 5.74 (4)
Strombine dehydrogenase (mU mg^−1^)	80.39 ± 13.06 (3)	61.63 ± 15.62 (3)	69.91 ± 9.62 (4)	110.15 ± 22.77 (4)
Alanopine dehydrogenase (mU mg^−1^)	336.54 ± 62.73 (4)	270.35 ± 130.97 (4)	196.56 ± 63.68 (4)	458.88 ± 86.61 (5)

**Notes.**

Values are mean ± standard error (N = number of organisms).

## Discussion

We have previously shown that GSH levels in the whole body of *B. solisianus* increase by about 60% in mussels exposed to air for 4 h during a low tide in the late afternoon (Moreira et al., 2021b). The concentration of markers of oxidative stress (TBARS and carbonyl protein) also increases during the low tide (Moreira et al., 2021b). On the other hand, in the current study, we found no changes in any biochemical variable measured in mussels of similar sizes from the same natural population collected in the next day, from the exact location and exposed to air or underwater. To understand the cause of this apparent discrepancy, we retrieved meteorological records and found that the first study (Moreira et al., 2021b) was conducted on a day of high global solar radiation incidence (15,381 kJ/m^2^). Although we did not consider the possible effect of solar UVR on the mussel’s redox metabolism in that previous study (Moreira et al., 2021b), here we provide observational results suggesting that UVR may have played a relevant role in the POS-response. Moreover, such activation of the POS mechanism in mussels exposed to air happened without a relevant influence of temperature.

During the early-morning low tide, when solar radiation was very low, the levels of oxidative stress markers (TBARS and carbonyl protein) and glutathione (as tGSH and GSH) were on baseline values on both sunny and cloudy days. These redox biomarkers remained at baseline levels on both days during high tide, lasting until the late afternoon. This high tide lasted for 9 h, after which aerial exposure happened again. Within 30 min of aerial exposure, the redox biomarkers were still unchanged. Levels of glutathione and markers of oxidative increased after 4 h of aerial exposure only on the sunny day. These results suggested the following observations: (i) redox biomarkers are not increased during aerial exposure alone, without intense UVR, since nothing happened in the early morning of both days and the evening of the cloudy day; (ii) solar UVR alone (either high or low intensity), without aerial exposure, cannot increase redox biomarkers after 7 h of continuous solar exposure to submerged mussels (at 2:45 PM on day 1 and 3:30 PM on day 2); (iii) Levels of redox biomarkers increased only in mussels that had been 9 h underwater on the sunny day, following more than 30 min of aerial exposure (since the short period of the low tide did not cause an increase in glutathione).

In this context, it is relevant to point out that collected mussels at high tide were only 1 to 2 ft underwater, with valves open. This would let solar UVR reach the animal’s soft tissue during high tide. Moreover, their location in the rock they were collected (at the top of it, see Picture in Supplemental Material) was in a position allowing solar radiation reach; good water transparency (see Methods) also contributed to that. On the other hand, when the shells are closed, during aerial exposure, the solar radiation cannot reach the internal tissues, since the shell of this species is fully opaque–it is translucid only in the larval stage (Cerrato, 2000; Monteiro-Ribas et al., 2006). Moreover, Moreira et al. (2021b) reported no gapping activity in this species during aerial exposure, precluding even minimal solar on the soft body inside shells during air exposure.

The combined effects of solar UVR and aerial exposure in mussel POS phenotype can be explained by increased RONS formation and Nrf2 activation due to functional hypoxia (Hermes-Lima et al., 2015; Moreira et al., 2017b; Geihs et al., 2020). Intracellular formation of RONS in diverse organisms can be induced by UVR in a complex and multifaceted signal networks (Dahms & Lee, 2010; Kim et al., 2011; Won et al., 2014; Häder et al., 2015; Fang et al., 2022) that possibly involves activation of NADPH oxidases and cyclooxygenases (Beak, Lee & Kim, 2004; Schuch et al., 2017). In mammalian cells, UVR activates redox-sensitive transcription factors, such as Nrf2 (Schafer et al., 2010; Knatko et al., 2015; Schäfer & Werner, 2015; Ikehata & Yamamoto, 2018), which stimulates the expression of endogenous antioxidants, including enzymes responsible for GSH biosynthesis (Schafer et al., 2010; Hermes-Lima et al., 2015). We recently proposed that UVR-induced antioxidant activation in many animal species–aquatic or terrestrial–works through a molecular mechanism that can be considered a POS-type response, similar to the hypoxia-induced POS phenotype (Geihs et al., 2020).

**Figure 5:**
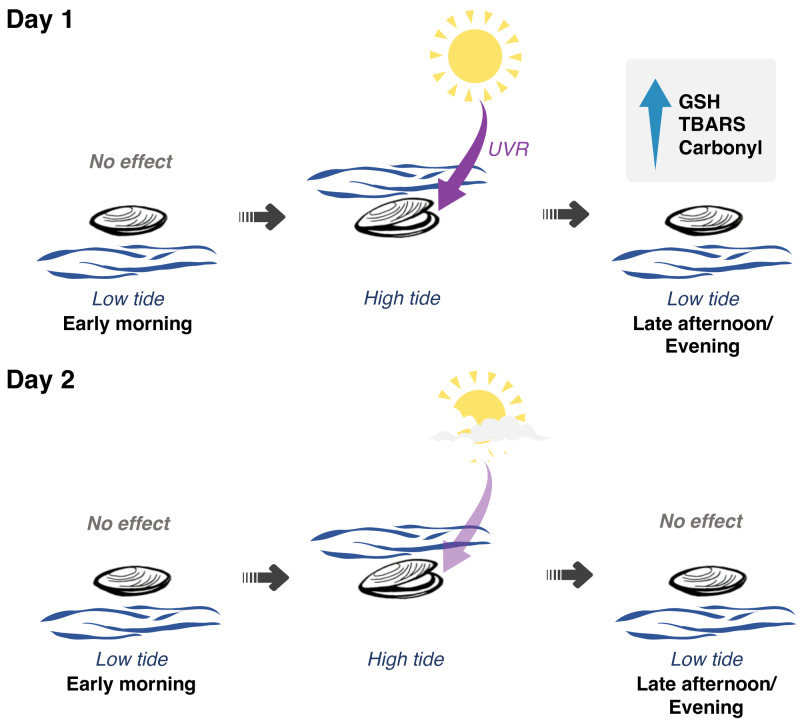
Interplay between solar radiation, air exposure and redox metabolism in *Brachidontes solisianus* mussels during the tidal cycle. Biochemical data of animals collected on two consecutive days, which differed from each other in terms of solar radiation revealed the possible role of ultraviolet radiation (UVR) in causing oxidative stress and triggering glutathione (GSH) synthesis. Air and water temperature, as well as the timing of air exposure and submersion, did not differ between days. Findings indicate that neither air exposure or UVR as a single stressor were able to disrupt redox balance. Of the four events of air exposure, only one, when animals were previously exposed to high solar radiation, was able to cause oxidative damage to lipids (TBARS) and proteins (carbonyl) and increase GSH levels.

The POS mechanism induced by UVR in mussels seems activated only in combination with functional hypoxia caused by the low tide and aerial exposure. In hypoxia-tolerant animals, internal hypoxic conditions should prompt increased mitochondrial RONS formation and, therefore, redox-activation of Nrf2, which could trigger the POS phenotype (Hermes-Lima et al., 2015; Moreira et al., 2017b). The increased lipid peroxidation levels corroborate the hypothesis of an augmented RONS generation and carbonyl protein in *B. solisianus* exposed to air and high solar UVR –none of this happens with low UVR levels happening during aerial exposure in the morning of both days nor in the late-afternoon plus evening of the cloudy day (see Table 4 and Fig. 5). On the other hand, due to increased GSH concentration, a higher antioxidant capacity might have limited the degree of oxidative stress. In other words, the rise in oxidative damage to mussel lipids and proteins in the evening on the sunny day could have been higher without the POS-response.

**Table 4:** Summary of biochemical changes in *Brachidontes solisianus* mussels during the tidal cycle. Animals were exposed to air twice (early morning and late afternoon) on each day in a total of four air exposure events.

**Condition**	**Glutathione**	**TBARS**	**Carbonyl**
*Day 1* ^a^			
Air exposure (early morning)	↔	↔	↔
Underwater	↔	↔	↔
Air exposure (late afternoon/evening)	↑	↑	↑
*Day 2*			
Air exposure (early morning)	↔	↔	↔
Underwater	↔	↔	↔
Air exposure (late afternoon/evening)	↔	↔	↔

**Notes.**

a Experimental conditions (Day 1) refer to results published in Moreira et al. (2021b).

### The transition from day 1 to day 2

It remains to be elucidated how increased levels of GSH and oxidatively damaged proteins and lipids from the evening of the sunny day decreased to basal levels in the morning of the next day? Several mechanisms might have contributed to this outcome, including repair and replacement (indirect repair) processes. Oxidized proteins, for example, are degraded by proteases to their amino acids, some oxidized others undamaged. Those undamaged amino acids are then used for a new synthesis of proteins, closing a recycling process. The lack of a known pathway that directly reverses protein-carbonyl modifications (Moskovitz & Oien, 2010) further supports the idea that oxidized proteins were repaired (directly or indirectly) from one day to another. A similar process also happens for oxidatively damaged lipids. Phospholipases, some with an augmented affinity for oxidized lipids (van Kuijk et al., 1987), hydrolyze these compounds, decreasing their levels. Other enzymes, such as glutathione peroxidase and glutathione transferases, act on lipid hydroperoxides and lipid peroxidation products, respectively. In the case malondialdehyde, aldehyde dehydrogenase and decarboxylase initiate their degradation to CO_2_ in a series of enzymatic steps (Ayala, Muñoz & Argüelles, 2014). Lastly, since oxidized lipids and proteins returned to basal levels in the early morning of day 2 in “our mussels”, the degree of oxidative damage to proteins, possibly caused by UVR exposure plus aerial exposure, did not overcome mussels’ repair and replacement capacity.

A comparable scenario was observed in *Perna perna* mussels exposed to air under laboratory conditions. After animals were exposed to air for 24 h, the levels of oxidative damage to DNA increased in the gills and digestive gland, and the degree of lipid peroxidation increased in the digestive gland (Almeida et al., 2005). Three hours after re-submersion, the levels of these oxidative stress markers returned to baseline levels in both tissues (Almeida et al., 2005). The occurrence of repair mechanisms is supported by the upregulation of proteins involved in repairing oxidative damage in the salt marsh mussel *Geukensia demissa* during recovery from air exposure (Fields et al., 2014). Other evidence of the upregulation of damage repair is the increase in the activity of GST in the digestive gland of *P. perna* mussels during recovery from air exposure (Almeida et al., 2005).

At least two processes might explain the decrease in GSH concentration from ∼0.2 mM to ∼0.1 mM in mussels during night-time, between the evening of the sunny day to the early morning of the cloudy day. The first is the consumption of glutathione in the repair mechanisms presented above. The conjugation of glutathione with exogenous and endogenous electrophiles, such as lipid peroxidation products, would remove it from the tGSH pool (*i.e.,* it does not simply oxidize it to GSSG). The second is the degradation of GSH to its corresponding amino acids or dipeptides. Two recently discovered enzymes are involved in cytosolic glutathione degradation in animals. They are the ChaC1 and ChaC2 enzymes, members of the gamma-glutamylcyclotransferases family. These enzymes degrade glutathione into 5-oxoproline and Cys-Gly (Kaur et al., 2017; Bachhawat & Yadav, 2018). The amino acids resulting from GSH degradation could, in turn, be used for new synthesis of proteins and peptides. Nevertheless, studying how enzymes involved in GSH biosynthesis and degradation regulate glutathione homeostasis and the POS-phenotype in mussels is a new challenge.

### POS mechanism induced by UVR

In our system, the peak of solar UVR (from about 10 AM to 3 PM) does not match the moment of increased variables of redox metabolism (glutathione, TBARS and carbonyl protein). When these biochemical variables increased the solar radiation had already decreased. However, the critical factor is the cumulative exposure to UVR (lasting 9 h, during which animals were underwater with valves open), which is maximal when the low tide starts. UVR triggers several cellular/biochemical events that increase RONS formation. In our hypothesis, the level of cellular stress able to induce oxidative damages and the POS response (increased GSH synthesis) is attained only by a further increase in RONS formation in aerial exposure. Such increase was recorded only after 4 h of low tide –it could have happened earlier than 4 h, but later than 30 min of aerial exposure (it is relevant to note the high variability in tGSH levels at 30 min of air exposure in the sunny day; 1/3 of the specimens had over 0.3 mM tGSH). Thus, in our setting neither of the abiotic events alone (UVR and aerial exposure) could induce significant changes in biochemical variables related to redox metabolism. They had to happen in synergism –one right after the other –to induce the POS phenotype.

### Temperature effect

One environmental variable that could have affected the redox metabolism in *B. solisianus* is temperature. The ability of high temperatures to influence the redox metabolism in tissues of aquatic animals is well-known. However, there is not a clear pattern of antioxidant responses to temperature, and higher temperatures do not necessarily induce oxidative stress nor activation of endogenous antioxidants in tissues of various aquatic invertebrate species, being a species-specific response (Matozzo et al., 2013; Vinagre et al., 2014; Jimenez et al., 2016; Kamyab et al., 2017; Moreira et al., 2017a; Grilo et al., 2018; Feidantsis et al., 2020). In our case, temperature oscillated minimally along the sampling hours of both days, varying from 23 °C to 26 °C, either in air or seawater. There was also no relevant difference in temperatures between sunny and cloudy days. In addition, temperatures peaked in mid-afternoon, when mussels were underwater with unchanged redox biochemical markers. Thus, the observed increased in redox markers in the evening if day 1 was probably unrelated to temperature.

### Ecological biochemistry field studies

Our main motivation to obtain data from animals collected directly from the field environment, with no intervention at all, is to take a glimpse at the biochemical response of animals in a real-world situation (Spicer, 2014). Data from neuroendocrine studies indicate that the results from animals kept in captivity might differ, in some cases drastically, from those obtained from field animals (Calisi & Bentley, 2009). Although completely natural field experiments, as done here, do not strictly control experimental conditions (*e.g.*, temperature, solar radiation, *etc*.) that might influence results, they are as close to reality one can get, providing valuable insights into the POS-mechanism in the actual environment (Spicer, 2014). On the other hand, it can be very hard to accurately reproduce the conditions of one experiment to another, given the large natural variations in the environment. Despite obtaining biochemical data for only two days of field work, this study managed to collected animals in a set of six different natural conditions with relevant characteristics and differences in terms of environmental variables. N ≥ 7 animals were collected for each of these groups to measure glutathione and oxidative stress markers. Six major natural conditions were covered: (i) early morning low tide, day 1; (ii) early morning low tide, day 2; (iii) high tide, day 1 (late morning plus afternoon); (iv) high tide, day 2 (late morning plus afternoon); (v) late afternoon plus evening low tide, day 1; and (vi) late afternoon plus evening low tide, day 2.

Although we, purposely, did not control every environmental variable as in lab research, we could compare animals exposed to a very similar tidal cycle (*i.e.,* temperature, time of exposure, duration) but with a tremendous (*i.e.,* 2.8-fold) difference in terms of global solar radiation. This is why we attribute the observed biochemical changes in oxidative stress markers and glutathione to a possible effect of UVR (POS mechanism induced by UVR). However, we cannot rule out the effects of other environmental factors whose data we do not have (*e.g.*, salinity, microbiota, and contaminants). But, for example, salinity is unlikely to have changed significantly from one day to another, since natural variations in salinity occur seasonally rather than on a daily basis in this region (Resgalla et al., 2007). As mentioned above, air and seawater temperatures were remarkably stable in the period of animal collection, and is unlikely to have played any role. Nevertheless, our hypothesis needs to be further tested under strictly controlled settings in the lab. Another effect we cannot ignore is any inter-day influence on the biochemical parameters. We do not know what happened the day before day 1 and we cannot exclude that the events occurring in day 1 affected the response on day 2. New field expeditions for the study the solar radiation in mussel redox variables would be desirable to strengthen our hypothetical explanations.

Finally, we calculated the possibility that all three redox indicators (glutathione, TBARS and carbonyl protein) become increased only during the evening low tide of day 1 (*i.e.,* in only one condition, out of six). This is a situation where air exposure happens after mussels had been exposed underwater to intense solar radiation for hours. To simplify calculations, we just tabulated the variables as “not increased” (summing “no significant change” and “decreased” in this category) or “increased”. So, the possibility that redox biomarkers are all increased in this particular condition (evening low tide of day 1) would be just 1 in 816, or 0.12%. Therefore, it is highly improbable that the increase in all three redox indicators happened by chance at “condition 3” (see Table 4).

### Controlled experiments *versus* observational biology

It is fundamental to differentiate the present observational results, also called natural experiments (Diamond, 1983), obtained from wild animal sampling, to those of strictly controlled laboratory studies, where researchers run a well-defined control group *versus* experimental ones. As stated in the Introduction section, almost all animal studies on the POS response were done in the lab, except for a small list, including a mussel work from Moreira et al. (2021b). When we performed the bivalve captures in 2014, we did not anticipate the possibility that solar UVR could play any role in the redox metabolism of mussels. This only became clear when we analyzed samples from another expedition-day when no changes were observed in any of the metabolic and redox variables. The sole difference between the two days of animal sampling was solar radiation. So, the present study is an attempt to rationalize this unpredicted observation. According to Jared Diamond, “*the obvious weakness of natural experiments is that the observer does not create a known difference between two situations, but must instead decide what difference between two existing situations is the salient one*.” (Diamond, 1983). In our case, it is solar radiation. It would have been better if this had started as a lab-controlled experiment. However, the trade-off for the inability to manipulate the wilderness is more realism (Diamond, 1986). Still, this proposed effect of UVR is actually a “side effect” of our search for the POS-mechanism in nature. Therefore, it is a must that such mechanism of “POS-UVR” be analyzed for other intertidal bivalve species.

## Conclusions

Although highly productive, coastal environments are characterized by wide fluctuations in several environmental parameters (*e.g.*, temperature, oxygen availability, salinity, pH, nitrogenous waste, hydrogen sulfide, and UVR) that often vary simultaneously (Leeuwis & Gamperl, 2022). The interaction effect between environmental stresses on the redox balance of bivalves has been demonstrated, for example, in *Callinectes sapidus* (temperature × hypoxia; (Garcia-Rueda et al., 2022), *Chlamys farreri* (temperature × air exposure; (Chen et al., 2007b), *Crassostrea hongkongensis* (salinity × hypoxia; (Wei et al., 2022), *Hyriopsis cumingii* (harmful algae × hypoxia; (Hu et al., 2015), *Mytilus coruscus* (pH × hypoxia; (Sui et al., 2017), and *Mya arenaria* (pH × CO_2_ × hypoxia; (Ouillon et al., 2021). In the case of solar radiation, although it is known that exposure to high levels of UVR leads to the mortality of intertidal invertebrates (Rawlings, 1996; Przeslawski, Davis & Benkendorff, 2005), its interaction with other environmental stressors, especially under natural conditions, in disturbing redox processes remains largely unexplored. Our study indicates that UVR may play a role as an interacting factor with another environmental variable (the tide cycle). However, more tests are necessary to confirm this in laboratory conditions, or in the field but with some control of key variables, such as shading mussels in clear sky days.

Finally, the present results suggest that air exposure under low solar radiation conditions is insufficient to trigger preparation for oxidative stress in *B. solisianus* in its natural habitat (Fig. 5). We propose that only when animals are previously exposed to high solar radiation does the air exposure-induced stress surpass a threshold that causes oxidative stress and triggers glutathione synthesis. The research has also evidenced the remarkable resilience of *B. solisianus* to endure the fluctuating conditions of the intertidal environment and its capacity to repair oxidative damage of biomolecules from one day to another. However, more research is needed to understand such repair mechanisms better, especially in natural conditions.

##  Supplemental Information

10.7717/peerj.15345/supp-1 Supplemental Information 1 The role of solar radiation and tidal emersion on oxidative stress and glutathione synthesis in mussels exposed to air Raw measurements and the output of all statistical analyses.

## Additional Information and Declarations

### Field Study Permissions

The following information was supplied relating to field study approvals (i.e., approving body and any reference numbers): This study was conducted under the license issued by ICMBio and SISBIO (permit number 20030–9).

### Data Availability

The following information was supplied regarding data availability: The raw measurements, as well as the output of all statistical analyses, are available in the Supplementary File.
